# Common Regulators of Lipid Metabolism and Bone Marrow Adiposity in Postmenopausal Women

**DOI:** 10.3390/ph16020322

**Published:** 2023-02-20

**Authors:** Dae-Yong Kim, Seong-Hee Ko

**Affiliations:** 1 CEO, N- BIOTEK, Inc., 402-803, Technopark, 655, Pyeongcheon-ro, Bucheon-si 14502, Gyeonggi-do, Republic of Korea; 2 Regenerative Medicine Research Team, N- BIOTEK, Inc., 104-706, Technopark Ssangyong 3Cha, 397, Seokcheon-ro, Bucheon-si 14449, Gyeonggi-do, Republic of Korea

**Keywords:** estradiol loss, dysregulated lipid metabolism, peroxisome proliferator-activated receptor-γ coactivator 1α, estrogen-related receptor alpha, bone marrow adiposity, bone loss, osteoporosis

## Abstract

A variety of metabolic disorders are associated with a decrease in estradiol (E2) during natural or surgical menopause. Postmenopausal women are prone to excessive fat accumulation in skeletal muscle and adipose tissue due to the loss of E2 via abnormalities in lipid metabolism and serum lipid levels. In skeletal muscle and adipose tissue, genes related to energy metabolism and fatty acid oxidation, such as those encoding peroxisome proliferator-activated receptor-γ coactivator 1α (PGC-1α) and estrogen-related receptor alpha (ERRα), are downregulated, leading to increased fat synthesis and lipid metabolite accumulation. The same genes regulate lipid metabolism abnormalities in the bone marrow. In this review, abnormalities in lipid metabolism caused by E2 deficiency were investigated, with a focus on genes able to simultaneously regulate not only skeletal muscle and adipose tissue but also bone metabolism (e.g., genes encoding PGC-1α and ERRα). In addition, the mechanisms through which mesenchymal stem cells lead to adipocyte differentiation in the bone marrow as well as metabolic processes related to bone marrow adiposity, bone loss, and osteoporosis were evaluated, focusing on the loss of E2 and lipid metabolic alterations. The work reviewed here suggests that genes underlying lipid metabolism and bone marrow adiposity are candidate therapeutic targets for bone loss and osteoporosis in postmenopausal women.

## 1. Introduction

Decreased estradiol (E2) levels are an important risk factor for dysregulated lipid metabolism [1]. Circulating E2 levels range from 2000 to 15,000 pg/mL in women of childbearing age but decrease to 10 pg/mL in postmenopausal women [2]. As a result of these rapid E2 changes, postmenopausal women are more likely to suffer from various metabolic disorders, such as abnormal lipid metabolism, visceral fat accumulation, and changes in fatty acid metabolism [1,3,4]. Additionally, surgical menopause is accompanied by severe dyslipidemia and a significant loss of bone density [5].

Lipid metabolism abnormalities due to reduced E2 not only increase the level of low-density lipoproteins (LDL-C) in the blood [6] but also lead to fat accumulation and abnormal fatty acid oxidation in skeletal muscle and adipose tissues [7]. In particular, genes related to energy metabolism and fatty acid oxidation, such as those encoding peroxisome proliferator-activated receptor-γ coactivator1α (PGC-1α) estrogen-related receptor alpha (ERRα), are down-regulated, resulting in fat accumulation [8,9]. Interestingly, these genes that are downregulated in skeletal muscle and adipocytes are also downregulated in bone marrow adipocytes (BMA) in an ovariectomized (OVX) model [10]. Furthermore, fat accumulation in the bone marrow [11] and fatty acid oxidation-related metabolism are downregulated, leading to bone loss and osteoporosis [10]. Bone marrow adipose tissue (BMAT) has been recognized as simply acting as a fat reservoir [12]. Postmenopausal E2 deficiency is associated with rapid bone loss, osteoporosis, and an increased risk of fracture based on recent studies of humans and OVX animal models and is accompanied by the abnormal accumulation of marrow adipose tissue (MAT) [11,13]. Treatment with E2 attenuates bone loss and decreases the amount of marrow fat [14,15].

Consequently, postmenopausal women tend to develop lipid abnormalities in the blood, increase fat accumulation in muscle, adipose tissue, and bone marrow, and increase obesity and adiposity [1,16]. In particular, when fat increases in the bone marrow due to a decrease in E2, BMD reduction, bone loss, and osteoporosis are likely to occur [16,17,18].

However, in this review, we took a holistic approach to changes in lipid metabolism in postmenopausal women regarding abnormal lipid metabolism in blood, muscle, adipose tissue, and bone marrow. The background of postmenopausal women’s mesenchymal stem cells (MSC), which are biased towards adipocyte differentiation and are prone to fat accumulation and bone marrow adiposity, is briefly summarized. Furthermore, genes involved in the E2 reduction in E2 in bone marrow, as well as skeletal muscles, and adipose tissue and commonly involved in fat metabolism regulation as well as and bone metabolism regulation were briefly described. Recent information on the mechanisms and epidemiological studies of bone marrow adiposity in postmenopausal women leading to BMD reduction, bone loss, and osteoporosis are briefly summarized. Genes that can control lipid metabolism in muscle and adipose tissue as well as in bone marrow could lead to the development of drugs that commonly regulate adiposity in muscle tissue, adipose tissue, and bone marrow in postmenopausal women.

## 2. Abnormal Blood Lipids and Fat Accumulation in Postmenopausal Women

### 2.1. Blood Lipid Abnormalities in Postmenopausal Women

Typically, women experience menopause spontaneously between the ages of 45 and 55 years due to a decrease in ovarian follicular activity [19]. In pregnancy, circulating E2 levels increase significantly from early pregnancy to delivery, ranging from 2000 to 15,000 pg/mL, whereas postmenopausal women exhibit a decrease in circulating E2 concentrations to 10 pg/mL [1,20]. These dramatic E2 changes predispose postmenopausal women to the development of cardiovascular diseases due to changes in lipid metabolism [2]. E2 is mainly produced in the ovaries using LDL cholesterol (LDL-C) as a substrate. However, when E2 synthesis declines with menopause, circulating LDL-C is no longer consumed for E2 synthesis and therefore remains in the systemic circulation [7]. The synthesis of E2 using LDL-C is reduced, resulting in elevated levels of LDL-C in the blood and dyslipidemia [1]. Postmenopausal women have an increased risk of metabolic syndrome symptoms, such as abdominal obesity, insulin resistance, dyslipidemia, hypertension, cardiovascular disease, and osteoporosis, due to high LDL-C levels [21]. For example, in association with surgical menopause, bilateral oophorectomy causes dyslipidemia and a marked reduction in bone mineral density (BMD) [22].

### 2.2. Fat Accumulation in Skeletal Muscle and Adipose Tissue in Postmenopausal Women

Many studies have reported that a decrease in E2 is related to fat accumulation in models of menopause [23,24]. In particular, animal models have demonstrated that E2 plays an essential role in fatty acid oxidation in mitochondria [4,25]. An aromatase (estrogen synthase) knockout mouse model is a menopause model created by the disruption of Cyp19, which encodes an enzyme that converts androgens to estrogens [26]. Because estrogen synthesis is blocked due to a lack of aromatase, estrogen is not synthesized in organs other than the gonads; accordingly, this model can be used to evaluate the roles of E2 [27]. Biochemical analyses of the aromatase knockout liver have reported the inhibition of mRNA expression and the activity of enzymes involved in fatty acid beta-oxidation [25]. In addition, OVX itself induces abdominal fat accumulation via increased gonadotropin-releasing hormone secretion in response to the E2 deficiency caused by OVX [23]. A recently identified aromatase pathway for visceral fat in the OVX model explains the mechanisms underlying visceral fat accumulation after menopause [26]. Using the OVX model, it has been demonstrated that the expression of all-trans-retinol 13,14-reductase (RETSAT), a gene related to lipogenesis, was increased and genes related to fatty acid oxidation were downregulated [4]. RETSAT is induced during adipocyte differentiation and is positively regulated by the transcription factor peroxisome proliferator-activated receptor [28], indicating that upregulated RETSAT is related to active fat accumulation in OVX mice. Kamei et al. have reported that the expression levels of energy expenditure-related genes and genes related to beta-oxidation in adipose tissue and skeletal muscle were downregulated under an OVX-induced E2 deficiency [8]. In particular, nuclear receptors and cofactors, such as peroxisome proliferator-activated receptor γ (PPARγ), PPARα, PGC-1α, PGC-1β, and ERRα, are downregulated at 2–4 weeks after OVX [8].

However, extensive studies of lipid metabolic alterations have been reported due to the accumulation of fat in adipose tissue and skeletal muscle after menopause. Regarding these lipid abnormalities, it is necessary to pay attention to the increase in bone marrow adiposity and its effect on bone homeostasis in postmenopausal women, which will be described in the next section.

## 3. Postmenopausal Bone Marrow Adiposity and Fatty Acid Metabolism Abnormalities

### 3.1. Basic Roles of Bone Marrow Adipose Tissue and the Loss of E2

Bone marrow is a semi-solid tissue found in the spongy portion of bones and is the main site of blood cell production. The human bone marrow produces about 500 billion blood cells per day, which enter the systemic circulation via permeable vasculature sinusoids within the bone marrow cavity [29]. Bone marrow consists of hematopoietic cells, BMAT, and supporting stromal cells [30]. All types of hematopoietic cells, including bone marrow and lymphoid lineages, are produced in the bone marrow, and MSCs, which can be isolated from the primary culture of the bone marrow matrix, can generate bone, adipose, and cartilage tissues [31]. In adults, the bone marrow is mainly located in the ribs, vertebrae, sternum, and pelvis [32]. Although the bone marrow of adult long bones is composed of abundant adipose tissue, the function of BMAs is largely unknown. Interestingly, BMAT constitutes more than 10% of the total fat mass in lean healthy humans and can account for up to 30% of total body fat, depending on peripheral fat mass [33].

For decades, BMAT has been considered a quiet bystander, filling the void left in the bone marrow following the age-related decline in hematopoiesis. However, the fat mass of bone marrow is closely correlated well with a loss in BMD as well as with osteoporosis, as in aging or menopause [34]. Since BMAs and osteoblasts originate from MSCs, they share a common ancestral lineage [35]. Furthermore, adipogenesis is considered a competitive process that interferes with osteoblastogenesis. Increased fat accumulation in bone marrow after menopause may result from abnormal lineage specification of MSCs [34]. The coexistence of BMA, mesenchymal stromal cells, hematopoietic cells, osteoblasts, and osteoclasts in the bone marrow creates a microenvironment that allows adipocytes to act directly on other cell types [36]. Moreover, most of the factors secreted by bone marrow and myeloid cells (ligands and antagonists of the WNT/β-catenin pathway, bone morphogenetic proteins, and so on) play a role in regulating the differentiation of MSCs into adipocytes or osteoblasts [34].

Interestingly, a recent study has revealed that E2 is important for the differentiation of MSCs into adipocytes, osteocytes, and chondrocytes [36]. E2 regulates the expression of adipogenesis-related transcription factors, such as PPARγ and C/EBPα, and estrogen acts as an epigenetic shifter in the regulation of H3K27 methylation to inhibit adipogenic differentiation of MSCs [36], which suggests that the loss of E2 can promote the adipose differentiation of MSCs. In addition to this, since estrogen, which regulates bone remodeling, also regulates the differentiation and activity of BMA, its rapid decrease during menopause increases bone marrow and fat differentiation and contributes to osteoporosis in postmenopausal women. In addition, Onji et al. demonstrated that the excessive expansion of BMAT is an important factor for bone loss and osteoporosis in postmenopausal women [37].

Taken together, E2 can regulate the adipogenic differentiation of MSCs, the activation of adipogenesis-related transcription factors, and the epigenetic modification of related genes. This provides a new approach to the regulation of fat metabolism and the treatment of bone loss and osteoporosis in postmenopausal women.

### 3.2. Menopause-Associated Fat Accumulation in Bone Marrow

Recent studies have reported a significant increase in bone marrow adiposity between the ages of approximately 55 and 65 years, that is, several years after menopause [38,39]. Additionally, a growing number of studies have shown that bone mass loss in OVX animal models is always accompanied by the abnormal accumulation of BMAT [11,40,41]. Estrogen supplementation results in an increase in bone mass and decrease in bone fat in estrogen-deficient humans and mice [15,42]. These results demonstrate that BMAT is associated with bone loss caused by estrogen loss. 

Since BMA and osteoblasts originate from a common precursor, MSCs, adipogenesis is considered a competitive process that interferes with osteoblastogenesis. A bias of MSCs towards the adipocyte lineage can directly impair the formation of bone-forming osteoblasts [43]. Estrogen plays an important role in maintaining the BMD by various mechanisms in cells such as osteoblasts, osteocytes, and osteoclasts. E2 binds to estrogen receptor alpha (ERα) and ERβ, and these receptors are involved in the regulation of E2 and bone metabolism [44]. Okazaki et al. demonstrated that E2 directly regulates the differentiation of benign stromal cells into osteoblastic and adipocyte lineages, resulting in a lineage shift to osteoblasts [45]. It has also been demonstrated that E2 inhibits adipogenesis in the bone marrow stromal cell line ST2 by inducing Transforming Growth Factor-β (TGF-β)-mediated connective tissue growth factor expression [46]. Studies have demonstrated the mechanism by which E2 promotes the preferential differentiation of human MSCs into osteoblasts rather than adipocytes [47].

In a study using cell-specific ERα knockout mice (αERKO), the absence of ERα in differentiated osteoblasts, osteocytes, and osteoclasts in mice resulted in greater bone mass deficits in trabecular bone of female mice but not in ERβ knockout mice [48]. This is consistent with the results of studies in which ERα and ERβ antagonize each other in bone and other tissues both in vitro and in vivo [44]. Gavin et al. performed bone marrow transplantation from donors expressing luciferase or green fluorescent protein into OVX mice and αERKO mice. Eight weeks after transplantation, the production of bone marrow-derived adipocytes was highest in OVX mice and αERKO mice, and the observed increases were attenuated in OVX mice by E2 add-back [49]. This means that the loss of E2 leads to the increased production of bone marrow-derived adipocytes.

High levels of follicle-stimulating hormone (FSH) stimulated by a persistent estrogen deficiency have also been shown to play a role in adipose differentiation. OVX mice treated with a polyclonal antibody of FSH exhibit a lower BMAT volume than that of controls subjected to increased FSH, indicating that increased FSH may promote adipose differentiation after menopause [50]. According to a cross-sectional study, AGES-Reykjavik Study of Older Adults, elevated serum FSH is associated with a lower bone mass, elevated bone marrow adiposity, and lower fat and lean mass in women [51].

Serum sclerostin (SOST) levels are negatively correlated with free E2 levels in postmenopausal women [52] and estrogen treatment in postmenopausal women reduces circulating SOST levels [53]. SOST is an osteocyte-derived protein and an important inhibitor of bone formation [54]. It functions by inhibiting the differentiation and activity of bone-forming osteoblasts by antagonizing the Wnt/β-catenin signaling pathway [55]. Ueland et al. found that an estrogen deficiency results in high bone marrow fat and concomitantly elevated SOST levels in postmenopausal women with osteoporosis [56]. In addition, SOST promotes adipogenesis of MSCs, proving that SOST, a Wnt inhibitor, can induce adipogenesis in 3T3-L1 cells, mouse ear- and BM-derived MSCs, and human BM-derived MSCs, demonstrating that a reduction of SOST significantly reduces BMAT formation [57].

### 3.3. Roles of PGC-1α and ERRα in Abnormal Lipid Metabolism in Bones of Postmenopausal Women

The loss of E2 is related to decreases in the expression of PGC-1α and ERRα in muscle and fat as well as in bone, which may affect fatty acid metabolism and BMD. In this section, these two genes are briefly reviewed, focusing on the mechanisms by which they contribute to fatty acid metabolism and osteogenesis.

#### 3.3.1. PGC-1α

The involvement of PGC-1 and ERRα in bone marrow obesity in postmenopausal women are particularly important among factors related to fatty acid oxidation in bone. 

The PGC-1 family include PGC-1α, PGC-1β, and PGC-related coactivators (PRCs), which interact with a full range of transcription factors involved in a variety of biological functions [58]. In other words, as a result of interacting as a transcription factor without directly binding to DNA itself, it was found that the resulting biological response was eventually regulated by PGC-1α. Therefore, this study will focus on PGC-1α [59]. 

PGC-1α, encoded by PPARGC1A, was first shown to mediate adaptive thermogenesis in brown fat [58]. Until now, PGC-1α is highly expressed in tissues with high energy requirements and is well known transcription factor that mediates thermogenesis and regulates antioxidant, anti-inflammatory, and energy metabolism [60]. In addition to this, PGC-1α plays a major role in protecting damage from oxidative stress and mediating the defense against inflammation by regulating the expression of antioxidant genes such as catalase, manganese superoxide dismutase, peroxiredoxin 3 and 5, uncoupling protein 2, thioredoxin 2, and thioredoxin reductase in mitochondria-rich cells [60]. Recently, Fahed et al. reported that dysregulation of PGC-1α activity in tissues can alter mitochondrial function and promote the accumulation of reactive oxygen species (ROS), thereby altering the metabolic properties of tissues and causing metabolic syndrome [60,61]. 

Furthermore, PGC-1α upregulates the expression of genes involved in red fibers, mitochondrial function, fatty acid oxidation, and branched-chain amino acid (BCAA) degradation in skeletal muscles to regulate the tricarboxylic acid cycle at the metabolite level. This suggests that PGC-1α plays crucial role in regulating energy metabolism [62]. Uldry et al. demonstrated that PGC-1α is required for the induction of thermogenic genes but not for brown fat differentiation in PGC-1α KO mouse model [63]. Additionally, PGC-1α can bind to various targets such as PPARα, PPARβ/δ, and PPARγ, and cooperate with the effector ERRα to regulate the expression of mitochondrial genes and indirectly contribute to fatty acid transport and utilization [64]. The ERRα binding site is located in the transcriptional control region of ERRα/PGC-1α-induced genes and contributes to the transcriptional response to PGC-1α [65]. It also upregulates the expression of several mitochondrial fatty acid oxidation pathway genes [66]. Notably, PGC-1α has the ability to stimulate peroxisomal activity such as oxidation of long- and very-long-chain fatty acids required for normal ossification [62,67]. PGC-1α overexpression promotes peroxisome production/function and fatty acid oxidation gene expression has been demonstrated in mouse brown adipocytes and liver tissue [68], as well as in human skeletal muscle [62].

Recently, important reports have been made regarding fatty acid oxidation and bone metabolism. Kashuwaha et al. reported that long-chain fatty acid oxidation is required for normal bone gain [67]. Additionally, not only has it been reported that mitochondrial long-chain fatty acid oxidation by osteoclasts is required for normal bone resorption [69], but it has also been demonstrated that fatty acid oxidation by osteoblasts is required for normal bone acquisition in a sex- and diet-dependent manner [70]. As such, long-chain fatty acid oxidation and oxidation of fatty acids are essential for normal ossification. To stimulate the activity of peroxisomes, therefore, PGC-1α, which plays this role, needs attention in relation to bone metabolism and fatty acid metabolism. In an OVX model, E2 reduction resulted in downregulation of PGC-1α in bone marrow as well as skeletal muscle and adipose tissue [10], which may lead to a decrease in the oxidation of long-chain fatty acids and fatty acids essential for normal ossification, so it may be suggested as one cause of the decrease in ossification after menopause.

Interestingly, Yu et al. reported that the level of PGC-1α decreases with aging in bone tissue [10]. Although PGC-1α acts as a co-activator involving the master regulator of adipogenesis, PPARγ, downregulation of PGC-1α in the OVX model resulted in the differentiation of mesenchymal stem cells into adipocytes rather than osteoblasts [10]. Yu et al. conditionally deleted PGC-1α in MSCs using Prx1-Cre (Prx1; PGC-1α f/f). Then, the effect of this deletion on osteoporotic bone loss and MAT accumulation in mice after OVX, mimicking postmenopausal osteoporosis, was evaluated [10]. In the case of OVX mice in which PGC-1α was specifically deleted in skeletal stem cells (Prx1; Pgc1af/f mice), the BMD (60%) and bone volume/total volume (54%) were significantly reduced compared to those in sham mice [10]. In the absence of PGC-1α after OVX, bone formation defects were exacerbated and bone mass was compromised; serum levels of osteocalcin were decreased, as did the rate and rate of bone formation. [10]. Concomitantly, OVX-associated PGC-1α deletion promoted estrogen deprivation-induced MAT accumulation [10], suggesting that when PGC-1α is downregulated due to the loss of E2, MSCs can differentiate into adipocytes and lead to MAT accumulation in postmenopausal women. In addition, the loss of PGC-1α markedly suppressed the expression of the PDZ-binding domain (TAZ), a transcriptional coactivator of Runx2 [10]. This suggests that PGC-1α may be a potentially important therapeutic target in the treatment of bone marrow adiposity, bone mineral density loss, osteoporosis, and skeletal muscle aging in postmenopausal women.

#### 3.3.2. ERRα

Recently, ESRRA has been a focus of research owing to its roles in the regulation of cell metabolism and function as well as its potential for the treatment of bone metabolism and bone homeostasis disorders [71,72]. The orphan nuclear estrogen receptor-related receptor alpha is expressed by osteoblasts and has clearly established functions in osteoprogenitor proliferation and differentiation [9]. In addition, ERRα expression is induced by E2 and increases fatty acid uptake/oxidation via increased mitochondrial replication, ATP generation, and attenuated ROS formation [73]. The loss of E2 in menopause results in the downregulation of fatty acid metabolism and ATP production and the accumulation of unoxidized fatty acid metabolites in tissues [74].

Of note, ERRα can regulate bone formation. It is highly expressed in ossification sites, promotes osteoblast differentiation, and activates osteopontin, a bone matrix protein [75,76]. The association between ESRRA and BMD was verified by a cross-sectional study. In an analysis of associations between BMD and ESRRA gene functional variants in 1335 premenopausal women, a statistically significant association was observed between ESRRA genotype and lumbar BMD [77]. Briefly, women with the long ESRRA genotype independently had a 3.9% (0.045 g/cm2) higher lumbar BMD than that of women with the short ESRRA genotype (*p* = 0.004) [77], suggesting that a single nucleotide polymorphism (SNP) in ESRRA is associated with an increased BMD in premenopausal women. Moreover, estrogen enhances ESRRA transcription in a dose-dependent manner in the early differentiation stage of osteoblast progenitor cells [9,78]. Progenitor cells with an ERRα deficiency show a tendency to differentiate into adipocytes via the increased expression of markers related to lipid metabolism, such as PPARγ [79]. Bonnelye et al. demonstrated that the down-regulation of ERRα expression by antisense treatment of rat calvaria cells not only inhibits osteogenesis but also increases adipocyte colony formation and changes the OPG/receptor activator of the nuclear factor kappaB ligand ratio; these findings indicate that ERRα may play a functional role in osteoblasts, adipocytes, osteoclasts, etc. in E2 deficiency diseases, such as osteoporosis, which is regulated by estrogen in bone [9]. The downregulation of ERRα due to the loss of E2 suppresses bone formation and may result in fat accumulation in MAT. 

Recently, ERRα has been reported to be involved in bone differentiation of MSCs. The expression of ERRα mRNA is significantly increased in the late stages of bone differentiation of human periodontal ligament stem cells (hPDLSCs) [80]. In addition, the transfection of cells with recombinant lentivirus-mediated miRNA targeting ERRα significantly suppressed the mRNA expression of genes related to alkaline phosphatase (ALP) activity, mineralization ability, and osteogenesis, including ALP, OCN (osteocalcin), runt-related transcript factor 2 (RUNX2), and Osteopontin, in hPDLSCs [80]. ERRα interacts with PGC-1α to enhance osteocalcin promoter activity and enhance transcriptional expression to promote osteogenesis [81]. OCN, the most abundant non-collagenous bone matrix protein, is produced specifically by osteoblasts and is suggested to regulate biological processes in multiple organs including bone and adipose tissue [82]. Taken together, the downregulation of ERRα due to the loss of E2 suggests that the osteogenic differentiation of MSCs is suppressed, and the interaction of ERRα with PGC-1α plays an important role in promoting osteogenesis.

### 3.4. Bone Marrow Adiposity and Bone Loss in Postmenopausal Women

A postmenopausal estrogen deficiency is associated with rapid bone loss and an increased risk of osteoporosis and fractures, which contributes to increased adipocytes in the bone marrow cavity [38,83]. In general, bone marrow adiposity increases in menopause women as aging progresses, and BMD and intra-bone fat production have an inverse relationship [18]. The lower the bone formation, the higher the fat production in the bone marrow [84]. In addition, mesenchymal cells extracted from the bone marrow of postmenopausal women with osteoporosis had more adipogenic differentiation characteristics compared to mesenchymal cells from the control group with normal bone mass [41]. Moreover, significant fat infiltration occurs in the bone marrow of rats after ovariectomy [41].

According to the Appendicular Muscle and Bone Extension Research Study, 312 postmenopausal women aged 60 to 85 years (75.4 ± 5.9 years, body mass index [BMI] 29.5 ± 5.7 kg/m^2^) had higher amounts of muscle fat, which was associated with a lower bone marrow density in 66% of the median tibia (B = 84.08 [27.56]), *p* = 0.002). Thus, bone marrow and muscle fat infiltration in postmenopausal women with osteoporosis were found to be correlated [16]. Additionally, the results of a recent cross-sectional study of 120 postmenopausal osteoporosis patients are also in the same line as above. In postmenopausal women, spinal bone marrow fat mass (BMA) was evaluated using magnetic resonance spectroscopy (MRS) and the correlation between BMD and BMA content was analyzed [17]. As a result, fat content (FC) was 47.0 (46.3 –78.8) and 46.4 (44.3–48.6), which were significantly high (*p* = 0.011). FC was significantly negatively correlated with BMD of the lumbar spine (Rho = −0.042; *p* < 0.001) and BMD of the hip (Rho = −0.64; *p* < 0.001). In the logistic regression model, FC was independently associated with osteoporosis (OR = 1.3; 95% CI 1.1–1.6) even after controlling for confounding factors (age, menopausal period, reproductive period, and body mass index) [17]. These results suggest that bone marrow adiposity can be an independent predictor of low bone mass in postmenopausal women. These properties have been clearly demonstrated using an animal model of ovarian resection and an estrogen receptor knockout model. A lower BMD was reported in the ERαKO model in the ErαKO model than in wild-type mice, and more fat cells were detected in the bone marrow of OVX rats than in sham-operated rats [85], which suggests that when E2 is lost after menopause, BMD decreases and fat accumulates in the bone marrow. Moreover, ERα is involved in lipid metabolism by regulating adipose triglyceride lipase and perilipin-mediated lipid metabolism and droplet size in bone marrow-derived MSCs and differentiated adipocytes in mouse femoral bone cultures [86]. Lipid droplets had a smaller diameter and were more abundant in adipocytes differentiated from ERαKO bone marrow than in ERβKO cells [86]. This suggests that E2 plays a protective role in regulating bone marrow fat accumulation via Erα.

Extensive studies have shown that E2 inhibits fat differentiation through Wnt signaling, a system important for bone metabolism [87,88,89]. It was reported that E2 suppressed the osteo-adipogenic trans-differentiation of MC3T3-E1 cells in a dose-dependent manner through canonical Wnt signaling pathway. In addition, MSC-derived osteoblasts in OVX mice exhibited higher trans-differentiation potential into adipocytic lineage compared to the sham group [84]. These findings observed both in vivo and in vitro, suggest that E2-deficient conditions may lead to disproportionate differentiation into adipocytes within the bone marrow.

Recently, it was reported that interactions among genetic factors contribute to osteoporosis in postmenopausal women. It has been reported that ERα and NFATc1 inhibit WNT5B in osteoblasts by binding to the non-coding SNP rs2887571 associated with bone density [90]. This is because WNT5B activates DVL2/3/RAC1/CDC42/JNK/SIN3A signaling and inhibits osteoblast differentiation via receptor tyrosine kinase-like orphan receptor 1/2 (ROR1/2), which inhibits β-catenin activity. This pathway may therefore be a target for osteoporosis therapeutics. 

Cross-sectional studies have reported that greater bone marrow adiposity is associated with a lower bone density and elevated prevalence of vertebral fractures [91,92]. However, in the Age Gene/Environment Susceptibility (AGES)-Reykjavik cohort study, a greater BMAT in older women resulted in a greater loss of trabecular bone in the spine and femoral neck and greater loss of spinal compressive strength [38], which suggests that high bone marrow adiposity may be a predictor of bone loss in older women.

Taken together, the loss of E2 is associated with rapid bone loss, osteoporosis, and an increased risk of fracture [93] as well as an increased BMA, as demonstrated by animal studies [11,83]. E2 plays a protective role in regulating bone marrow fat accumulation via ERα [86] and that genetic factors work together in postmenopausal osteoporosis [90]. Additionally, epidemiologic studies have also demonstrated that greater bone marrow adiposity predicts bone loss in older women [38]. These findings suggest that a comprehensive understanding of the relationship between BMAT expansion and lipid bone metabolism under the loss of E2 can provide new therapeutic targets for postmenopausal osteoporosis.

## 4. Conclusions

The menopause-induced E2 deficiency may disrupt lipid metabolism. It can cause excessive fat accumulation in muscle and adipose tissue of the musculoskeletal system and lipid abnormalities in the blood. In addition, fat accumulation is also found in the bone marrow, and genes involved in energy metabolism and fatty acid metabolism (e.g., PGC-1α and ERRα) are commonly downregulated. Excessive lipid accumulation in the bone marrow can cause rapid bone loss, osteoporosis, and an increased risk of fractures in postmenopausal women. The loss of E2 causes dysregulated lipid metabolism, including fat accumulation and the inhibition of fatty acid beta oxidation in skeletal muscle, adipose tissue, and bone marrow, associated with bone metabolism. In addition, bone marrow adiposity is a cause of bone loss and osteoporosis in postmenopausal women, along with genetic factors. These lipid metabolism abnormalities as well as bone marrow adiposity and osteoporosis are involved, in part, in the same genetic pathways that regulate energy metabolism and fatty acid oxidation. For example, PGC-1α and ERRα, which commonly influence body fat, visceral fat accumulation, and bone marrow adiposity in postmenopausal women, can be used as new therapeutic targets for bone loss and osteoporosis as well as abnormal lipid metabolism. If a drug that simultaneously modulates PGC-1α and ERRα is developed, a new drug that exhibits multiple functions for preventing bone marrow adiposity, improving bone mineral density, and reducing osteoporosis as well as improving systemic lipid metabolism in postmenopausal women can be presented.

## Data Availability

Data sharing not applicable.
